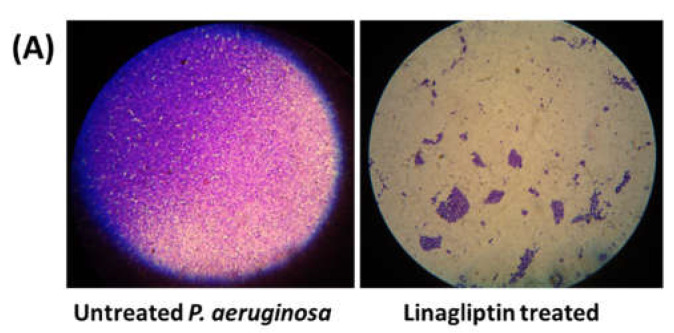# Correction: Khayat et al. Hiring of the Anti-Quorum Sensing Activities of Hypoglycemic Agent Linagliptin to Alleviate the *Pseudomonas aeruginosa* Pathogenesis. *Microorganisms* 2022, *10*, 2455

**DOI:** 10.3390/microorganisms13112415

**Published:** 2025-10-22

**Authors:** Maan T. Khayat, Tarek S. Ibrahim, Khaled M. Darwish, Ahdab N. Khayyat, Majed Alharbi, El-Sayed Khafagy, Mohamed A. M. Ali, Wael A. H. Hegazy, Hisham A. Abbas

**Affiliations:** 1 Department of Pharmaceutical Chemistry, Faculty of Pharmacy, King Abdulaziz University, Jeddah 21589, Saudi Arabia; 2 Department of Medicinal Chemistry, Faculty of Pharmacy, Suez Canal University, Ismailia 41522, Egypt; 3 Department of Pharmaceutics, College of Pharmacy, Prince Sattam Bin Abdulaziz University, Al-Kharj 11942, Saudi Arabia; 4 Department of Pharmaceutics and Industrial Pharmacy, Faculty of Pharmacy, Suez Canal University, Ismailia 41552, Egypt; 5 Department of Biology, College of Science, Imam Mohammad Ibn Saud Islamic University, Riyadh 11432, Saudi Arabia; mamzaid@imamu.edu.sa; 6 Department of Biochemistry, Faculty of Science, Ain Shams University, Abbassia, Cairo 11566, Egypt; 7 Department of Microbiology and Immunology, Faculty of Pharmacy, Zagazig University, Zagazig 44519, Egypt; 8 Department of Pharmaceutical Sciences, Pharmacy Program, Oman College of Health Sciences, Muscat 113, Oman

**Error in Figure**

In the original publication [1], there was a mistake in Figure 2A Linagliptin inhibits biofilm formation as published. Miss-insertion of Light microscope image of biofilm formation. The corrected Figure 2A appears below. The authors state that the scientific conclusions are unaffected. This correction was approved by the Academic Editor. The original publication has also been updated.