# Supplementary material for: Hiring of the Anti-Quorum Sensing Activities of Hypoglycemic Agent Linagliptin to Alleviate the Pseudomonas aeruginosa Pathogenesis
Source: Microorganisms. 2022 Dec 12;10(12):2455. doi: 10.3390/microorganisms10122455 (PMC9783625; doi:10.3390/microorganisms10122455)
Supplement: Supplementary file 1 [file microorganisms-10-02455-s001.zip › microorganisms-2020841-supplementary.pdf]

"Supplementary Data"

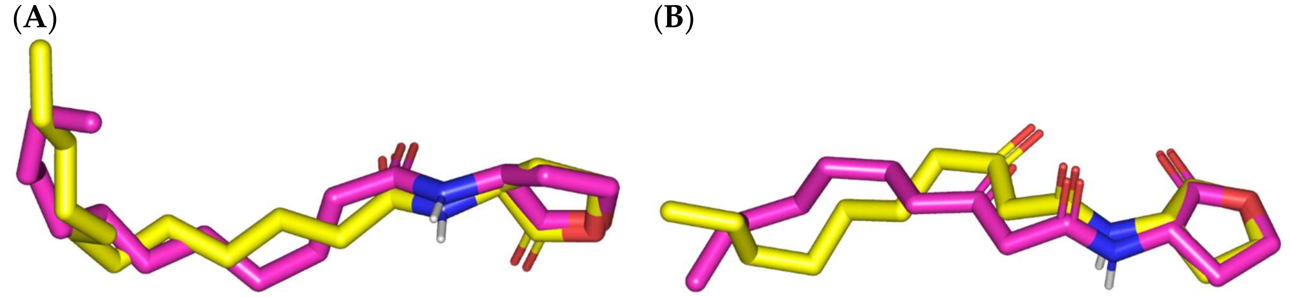

Figure S1. Superimposing the co-crystallized (magenta sticks) and redocked (yellow sticks) ligands at *P. aeruginosa* quorum-sensing transcription proteins QscR (**A**) and LasR (**B**) for validating the adopted directed docking protocol.

Table S1. Descriptive *P. aeruginosa* LasI-ligand binding interactions via docking protocol

Compound	Ligand-target interaction description<br>[Type; Length (Å); Angle (°); Binding Residues]
**LIN**	H-bond ; 2.4 **Å** ; 130 ° ; Arg30<br>H-bond ; 1.9 **Å** ; 147 ° ; Arg30<br>H-bond ; 2.2 **Å** ; 121 ° ; Thr142<br>H-bond ; 3.4 **Å** ; 141 ° ; Thr144<br>π-H interaction ; 2.3 **Å** ; Trp33<br>π-H interaction ; 1.9 **Å** ; Trp69
**TZD-C8**	H-bond ; 1.8 **Å** ; 123 ° ; Arg30<br>H-bond ; 3.4 **Å** ; 132 ° ; Ile170

Table S2. Descriptive *P. aeruginosa* QscR-ligand binding interactions via docking protocol

Compound	Ligand-target interaction description<br>[Type; Length (Å); Angle (°); Binding Residues]
**LIN**	Polar ; 2.6 **Å** ; Tyr66<br>Polar ; 2.8 **Å** ; Asp75<br>H-bond ; 2.8 **Å** ; 120 ° ; Ser38<br>H-bond ; 2.7 **Å** ; 127 ° ; Gly40<br>H-bond ; 3.0 **Å** ; 139 ° ; Ser129<br>π-π interaction ; 4.3 **Å** ; Phe54<br>π-H interaction ; 3.4 **Å** ; Tyr58<br>π-H interaction ; 3.5 **Å** ; Tyr66<br>π-H interaction ; 3.6 **Å** ; Trp102<br>van der Waals ; 4.6 **Å** ; Arg42 sidechain Cβ
**Q9**	H-bond ; 2.8 **Å** ; 146 ° ; Ser38<br>H-bond ; 3.2 **Å** ; 128 ° ; Trp58<br>H-bond ; 2.0 **Å** ; 161 ° ; Asp75<br>π-π interaction ; 3.9 **Å** ; Phe54<br>π-H interaction ; 3.5 **Å** ; Tyr66

Table S3. Descriptive *P. aeruginosa* LasR-ligand binding interactions via docking protocol

**Compound**	**Ligand-target interaction description**<br>**[Type; Length (Å); Angle (°); Binding Residues]**
**LIN**	Polar ; 3.4 **Å** ; Ser129<br>H-bond ; 3.1 **Å** ; 146 ° ; Tyr56<br>H-bond ; 2.3 **Å** ; 147 ° ; Trp60<br>H-bond ; 2.4 **Å** ; 146 ° ; Asp73<br>π-H interaction ; 4.0 **Å** ; Tyr47<br>π-H interaction ; 3.4 **Å** ; Tyr56
**Q9**	H-bond ; 1.9 **Å** ; 158 ° ; Tyr56<br>H-bond ; 1.7 **Å** ; 168 ° ; Asp73<br>H-bond ; 2.7 **Å** ; 126 ° ; Tyr93<br>H-bond ; 1.7 **Å** ; 163 ° ; Ser129<br>π-H interaction ; 4.2 **Å** ; Tyr47
